# Immunization with virus-like particles conjugated to CIDRα1 domain of *Plasmodium falciparum* erythrocyte membrane protein 1 induces inhibitory antibodies

**DOI:** 10.1186/s12936-020-03201-z

**Published:** 2020-03-30

**Authors:** Charlotte Harmsen, Louise Turner, Susan Thrane, Adam F. Sander, Thor G. Theander, Thomas Lavstsen

**Affiliations:** 1 Centre for Medical Parasitology, Department of Immunology and Microbiology (ISIM), Faculty of Health and Medical Sciences, University of Copenhagen, Copenhagen, Denmark; 2 Department of Infectious Diseases, Copenhagen University Hospital (Rigshospitalet), Copenhagen, Denmark

**Keywords:** Malaria, *Plasmodium falciparum*, Vaccine, Virus-like particle, Antigenic diversity, PfEMP1, CIDRα1

## Abstract

**Background:**

During the erythrocytic cycle, *Plasmodium falciparum* malaria parasites express *P. falciparum* Erythrocyte Membrane Protein 1 (PfEMP1) that anchor the infected erythrocytes (IE) to the vascular lining of the host. The CIDRα1 domain of PfEMP1 is responsible for binding host endothelial protein C receptor (EPCR), and increasing evidence support that this interaction triggers severe malaria, accounting for the majority of malaria-related deaths. In high transmission regions, children develop immunity to severe malaria after the first few infections. This immunity is believed to be mediated by antibodies targeting and inhibiting PfEMP1, causing infected erythrocytes to circulate and be cleared in the spleen. The development of immunity to malaria coincides with acquisition of broad antibody reactivity across the CIDRα1 protein family. Altogether, this identifies CIDRα1 as an important vaccine target. However, the antigenic diversity of the CIDRα1 domain family is a challenge for vaccine development.

**Methods:**

Immune responses in mice vaccinated with Virus-Like Particles (VLP) presenting CIDRα1 antigens were investigated. Antibody reactivity was tested to a panel of recombinant CIDRα1 domains, and the antibodies ability to inhibit EPCR binding by the recombinant CIDRα1 domains was tested in Luminex-based multiplex assays.

**Results:**

VLP-presented CIDRα1.4 antigens induced a rapid and strong IgG response capable of inhibiting EPCR-binding of multiple CIDRα1 domains mainly within the group A CIDRα1.4–7 subgroups.

**Conclusions:**

The study observations mirror those from previous CIDRα1 vaccine studies using other vaccine constructs and platforms. This suggests that broad CIDRα1 antibody reactivity may be achieved through vaccination with a limited number of CIDRα1 variants. In addition, this study suggest that this may be achieved through vaccination with a human compatible VLP vaccine platform.

## Background

Malaria caused by *Plasmodium falciparum* is still a leading disease accounting for considerable under-five mortality in sub-Saharan Africa. In areas of moderate to high transmission intensity, severe life-threatening malaria mainly affects infants and toddlers, as immunity against severe disease is acquired at a young age after a few malaria episodes [1–3]. Young children who have acquired immunity to severe infections are still susceptible to less severe malaria episodes and immunity against these gradually forms during childhood [4]. This development of immunity can be explained by a gradual acquisition of IgG against variable polymorphic proteins expressed on the surface of infected erythrocytes [5–9]. Members of the *P. falciparum* Erythrocyte Membrane Protein 1 (PfEMP1) family are considered main targets of this immunity. These proteins are anchored in the erythrocyte membrane exposing their large N-terminal to engage with receptors on endothelial cells (reviewed in [10]). This permits an effective sequestration of infected erythrocytes to the endothelial lining and allows these cells to escape blood flow and splenic filtration. On the erythrocyte surface, PfEMP1 is accessible to antibodies functionally inhibiting the binding between the infected erythrocyte and the endothelium. These antibodies are thought to be important mediators of immunity [11–14].

Each parasite genome contains 50–60 PfEMP1encoding *var* genes, which differ in sequence within and between parasites, and of which each parasite will express only one during the erythrocytic cycle [15]. Despite their extensive sequence diversity, the distribution of different PfEMP1 types is similar in all parasites [16]. PfEMP1s are composed of two to nine Duffy Binding-like (DBL) and cysteine-rich interdomain region (CIDR) domains, and in most PfEMP1 the second domain from the N-terminus is a CIDR domain [16]. These domains have diversified to confer binding to either endothelial protein C receptor (EPCR) (CIDRα1 domains) [17], CD36 (CIDRα2–6 domains), or unknown receptors (CIDRβ/γ/δ domains). These mutually exclusive binding phenotypes are maintained by chromosomal separation of the encoding genes, with so-called group B and C *var* genes encoding CD36-binding PfEMP1 and group A genes encoding EPCR-binding PfEMP1 and CIDRβ/γ/δ-domain PfEMP1. In addition, most parasites carry one to three variants of the so-called DC8 group B/A chimeric *var* genes also encoding EPCR-binding PfEMP1 [18].

Numerous studies have linked EPCR-binding parasites, or parasites expressing CIDRα1-PfEMP1, with development of severe malaria in including when it is presenting as cerebral malaria and severe anemia. No other PfEMP1 domain is consistently associated with severe malaria pathology [19–21]. However, CIDRα1-PfEMP1 are large multi-domain molecules, and it is likely that endothelial receptor-interactions of some accompanying domains act in concert with EPCR-binding to promote parasite survival. For example, current evidence suggests that supplementary binding to ICAM1 or HABP1 is associated with EPCR binding by group A [21, 22] and B/A PfEMP1 [23], respectively. PfEMP1 binding to EPCR abrogates EPCR-mediated conversion of protein C to activated protein C, which promotes a pro-inflammatory state of the endothelium prone to thrombin-induced expression of endothelial cell adhesion molecules including the Intercellular Adhesion Molecule 1 (ICAM1) [24, 25].

All CIDRα1 domains adopt a similar fold in order to bind EPCR with high affinity [26]. The EPCR binding mechanism of the CIDRα1 domain mimics that of activated protein C to EPCR, indicating that an evolutionary stalemate has been reached, in which neither the parasite or human protein can mutate its basic structure without compromising its vital function. However, immune selection pressure has imposed extensive sequence diversity across surface exposed amino acids on the CIDRα1 domain, albeit limited to variation maintaining structure and the physio-chemical properties of the amino acids directly interacting with EPCR. As a result, EPCR-binding CIDRα1 sequence variants distribute into six subgroups, CIDRα1.1 and CIDRα1.4–1.8, of which CIDRα1.1 and 1.8 are found in group B/A PfEMP1 and the rest among group A PfEMP1. The EPCR-binding site is essentially comprised of a ~ 25 amino acid kinked alpha-helix structure protruding from a core triple helix structure [26]. Across the 19 kDa minimal binding domain of CIDRα1, sequences are pairwise 40–100% identical. The average sequence identity within CIDRα1 subgroups (e.g. 1.1) is 69–72%, but short amino acid stretches of high similarity are shared across members of different subtypes [26].

Experimental immunization of mice and rats with single or multiple soluble CIDRα1 proteins, or adenovirus inducing in vivo secretion of CIDRα1 protein, readily elicit EPCR binding-inhibitory IgG to cognate antigens, as well as to CIDRα1 variants of the same CIDRα1 subtype as the used immunogen [27, 28]. The most heterogeneous of CIDRα1 subtypes is the CIDRα1.4 and CIDRα1.7 domains, to which it appears to be particularly difficult to elicit cross-ractive antibodies. Vaccines presenting antigens on Virus-Like Particles (VLP) have been shown to induce high tittered, long lasting functional IgG responses in humans [29, 30] and can drive immune responses even to weakly immunogenic or self-antgens [31, 32]. We, and others, have recently developed a technique that allows effective coupling of complex proteins to pre-formed VLP and demonstrated that the IgG response to the proteins coupled is potent, functional, and durable even with self-antigens [31, 33, 34]. Here, VLP-CIDRα1.4 vaccines adjuvanted with aluminum hydroxide elicited stronger immune responses than a similar soluble CIDRα1 vaccine in Freund’s incomplete adjuvant, and antibodies reactive and functionally inhibitory across group A CIDRα1 variants was elicited from immunization with a single CIDRα1 antigen variant.

## Methods

### Recombinant protein and VLP production

Proteins were produced in baculovirus-infected High Five cells as previously described [35]. CIDRα1 proteins derived from the HB3var03 PfEMP1 sequence were genetically fused with the SpyCatcher [36] protein sequence at the N-terminal or C-terminal and a Strep-tag II sequence in the opposite terminal (total size 32 kDa): catCIDRα1.4 (SpyCatcher-CIDRα1.4-Strep-tag II), and CIDRα1.4cat (Strep-tag II-CIDRα1.4-SpyCatcher).

SpyT-VLPs formed by 180 capsid units of the Acinetobacter bacteriophage AP205 and displaying one SpyTag per capsid unit, were produced as previously described [31]. In brief, the SpyTag sequence (AHIVMVDAYKPTK) was fused to the N-terminus of each capsid using a flexible linker (GSGTAGGGSGS). The construct was expressed in *E. coli* One Shot BL21 StarTM (DE3) cells (Thermo Scientific) and purified by ultracentrifugation using an OptiprepTM density gradient (Sigma). Naked VLP formed from AP205 capsids with no SpyTag were produced using the same method.

**Fig. 1:**
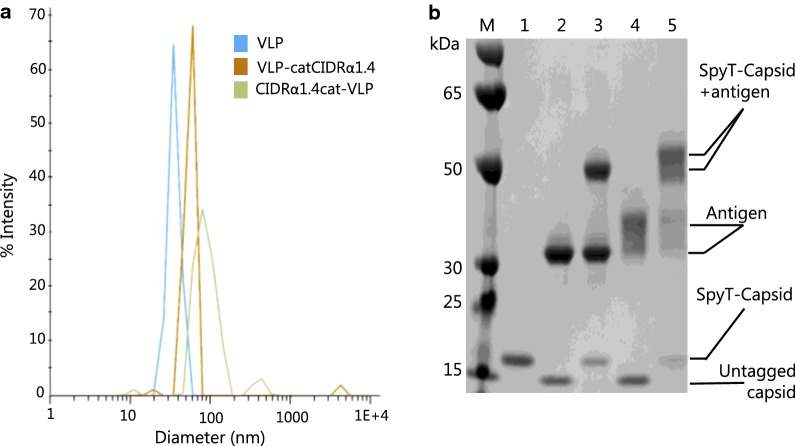
Characterization of VLP-antigen conjugated vaccines.** a** Dynamic light scattering (DLS) showing SpyT-VLP (blue) with an average size of 37 nm (12.18% polydispersity, Pd). After coupling VLP to catCIDRα1.4 (brown) or CIDRα1.4cat (green) the size increases to 56 nm (12.19%Pd) and 88 nm (27.83 %Pd), respectively.** b** SDS-gel loaded with catCIDRα1.4 or CIDRα1.4cat antigens (32kDa) mixed with SpyT-VLP (16kDa) or naked VLP (8kDa): 1. SpyT-VLP; 2. Naked VLP + catCIDRα1.4; 3. SpyT-VLP + catCIDRα1.4; 4. Naked VLP + CIDRα1.4cat; 5. SpyT-VLP + CIDRα1.4cat

Assembled SpyTagged VLP and soluble SpyCatcher-fused CIDRα1.4 antigen were mixed at a 1:1 molar ratio (VLP capsid per antigen) and incubated over night at 4 ℃, forming a ~ 48 kDa band on SDS-PAGE under reducing conditions (Fig. 1). Uncoupled antigen was not removed from the vaccine before administration.

Dynamic light scattering (DLS) was performed as previously described [31]. Briefly, the distribution of VLP particle sizes was acquired at 658 nm, 25  ℃ (WYATT Technology, DynaPro NanoStar). Each VLP preparation was measured twice with 20 runs and the estimated diameter of the main particle population and the percent polydispersity (%Pd) was calculated.

### Mice and immunizations

Female BALB/c mice (Taconic, Denmark), 7–8 weeks old, were immunized by intramuscular injection three times, at 3-week intervals. The mice were given 5 µg CIDRα1.4 antigen per mouse per immunization, administered as either bound to 2.5 µg SpyTag-VLP or without any VLP. In total 16 mice were distributed between the four groups: VLP-catCIDRα1.4 (five mice), CIDRα1.4cat-VLP (five mice), catCIDRα1.4 (three mice), and CIDRα1.4cat (three mice). VLP-coupled antigen was administered with Alhydrogel (2%) (Brenntag) added to the vaccine formulation 1 h prior to immunization, whereas antigens with no VLP was administered with Freund’s incomplete adjuvant (1:1, W/W). Serum was collected 2 weeks after each immunization. Total IgG was purified using GammaBind Plus Sepharose (BD Biosciences) according to the manufacturer’s protocol.

### ELISA analyses

Serum levels of antigen specific IgG were measured as previously described [31]. In brief, plates were coated with 5 µg/mL 30 kDa HIS-tagged HB3var03 CIDRα1.4 protein [28]. The wells were blocked with 1% BSA buffer. Mouse serum was added in a series of three-fold dilutions starting from 1:50. Secondary antibody (HRP-conjugated anti mouse IgG (Life Technologies, Denmark)) was diluted 1:3000 and color reactions were developed for 7 min by adding o-phenylenediamine substrate.

### IgG reactivity and EPCR-binding inhibition of diverse CIDRα1 domains

IgG reactivity to a panel of 43 recombinant CIDRα1 domains coupled to Luminex microspheres was measured as in [37]. In short, serum was diluted 1:80, and IgG reactivity was detected using secondary phycoerythrin (PE)-conjugated antibody diluted to 1:3000. For assessing EPCR-binding inhibition, purified IgG from each mouse within a vaccine group was pooled and incubated at 0.25 mg/mL IgG with the CIDRα1-conjugated Luminex microspheres for 30 min at room temperature. After washing with standard Luminex buffers, microspheres were incubated with 4 µg/mL biotinylated recombinant EPCR for 30 min at room temperature. EPCR-binding was detected using PE-conjugated streptavidin. The CIDRα1 protein variants with low binding to EPCR when coupled to microspheres were excluded from the inhibition study leaving 34 variants tested.

### Data analysis

Data were analysed using Microsoft Excel and STATA (StataCorp). Non-parametric statistical tests was used as indicated in the result section. IgG titers were calculated as area under curve using STATA14 using the pkexamine command using dilution as time parameter over 11 dilution steps, ELISA OD as concentration parameter and the trapezoidal rule.

### Protein sequences

#### catCIDRα1.4 (SpyC-HB3var03-Strep-Tag II) (linkers in lower case)

GAMVDTLSGLSSEQGQSGDMTIEEDSATHIKFSKRDEDGKELAGATMELRDSSGKTISTWISDGQVKDFYLYPGKYTFVETAAPDGYEVATAITFTVNEQGQVTVNGKATKGDAHIggsKITSFDEFFDFWVRKLLIDTIKWETELTYCINNTDVTDCNKCNKNCVCFDKWVKQKEDEWTNIMKLFTNKHDIPKKYYLNINDLFDSFFFQVIYKFNEGEAKWNELKENLKKQIASSKANNGTKDSEAAIKVLFNHIKEIATICKDNNTNEGrtgWSHPQFEK.

#### CIDRα1.4cat (Strep-tag-II-HB3var03-SpyC) (linkers in lower case)

WSHPQFEKrtgKITSFDEFFDFWVRKLLIDTIKWETELTYCINNTDVTDCNKCNKNCVCFDKWVKQKEDEWTNIMKLFTNKHDIPKKYYLNINDLFDSFFFQVIYKFNEGEAKWNELKENLKKQIASSKANNGTKDSEAAIKVLFNHIKEIATICKDNNTNEGggsGAMVDTLSGLSSEQGQSGDMTIEEDSATHIKFSKRDEDGKELAGATMELRDSSGKTISTWISDGQVKDFYLYPGKYTFVETAAPDGYEVATAITFTVNEQGQVTVNGKATKGDAHI.

## Results

### Production VLP-based CIDRα1 vaccines

Two recombinant CIDRα1 proteins both derived from the HB3var03 PfEMP1 CIDRα1.4 sequence were produced with a SpyCatcher protein sequence in either the N-terminal or the C-terminal (catCIDRα1.4 and CIDRα1.4cat, respectively) (Fig. 1). The recombinant proteins were coupled to bacteriophage AP205 SpyTag-VLP particles. These particles form spontaneously from 180 AP205 capsid proteins, each with a genetically fused SpyTag in the N-terminus. By mixing a recombinant SpyCatcher-fused CIDRα1.4 protein with the pre-formed VLP, the SpyTags and SpyCatchers formed covalent bonds, resulting in VLP coupled with CIDRα1.4 antigen (Fig. 1).

### Analysis of the IgG reactivity to the CIDRα1 domain used as immunogen

The two VLP based CIDRα1.4 vaccines and two vaccines containing the corresponding soluble proteins were administered by intramuscular injection to BALB/c mice over three immunizations with 3-week intervals. Two groups of five mice received catCIDRα1.4 or CIDRα1.4cat displayed on VLP in aluminum hydroxide and two groups of three mice received catCIDRα1.4 or CIDRα1.4cat without VLP but with Freund’s incomplete adjuvant.

**Fig. 2:**
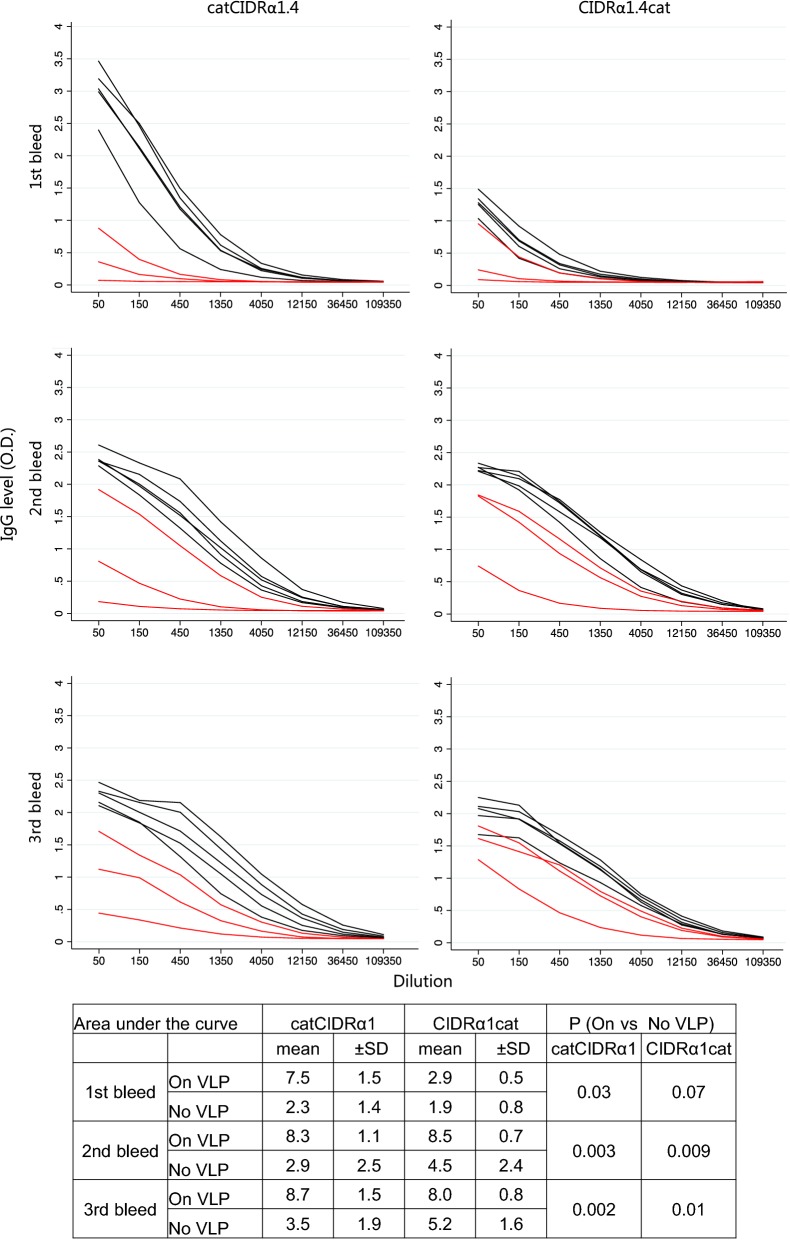
Anti-CIDRα1.4 IgG. ELISA titers elicited in mice vaccinated with catCIDRα1.4 or CIDRα1.4cat. Each line represents the titration of serum from a mouse. Black lines indicate serum from mice immunized with CIDRα1.4-VLP in aluminum hydroxide. Red lines indicate mice immunized with soluble CIDRα1.4 in Freund’s incomplete adjuvant. Table shows titers calculated as area under the curve for each vaccine group (mean ± standard deviation), P values from t-tests

Serum collected 2 weeks after each of the three immunizations was assessed for induction of IgG binding to cognate HB3var03 CIDRα1.4 domains (without the SpyCatcher tag of the immunogens) in ELISA. The CIDRα1.4-VLP vaccines in aluminum hydroxide induced a faster and stronger IgG response against the cognate HB3var03 CIDRα1.4 antigen than the vaccines with soluble antigen in Freund’s incomplete adjuvant (Fig. 2). The effect was most pronounced after the first immunization (Fig. 2, 1st bleed).

### Analysis of IgG reactivity to sequence diverse CIDRα1 domain variants

**Fig. 3:**
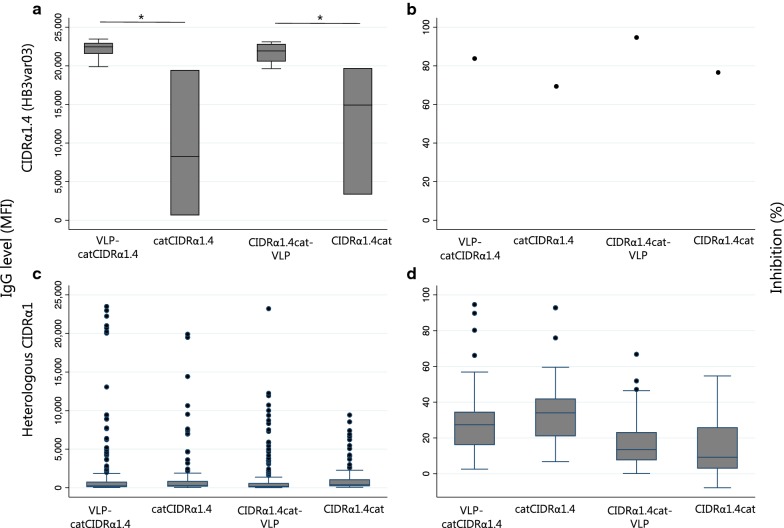
Antibody reactivity and EPCR-binding inhibition in Luminex. **a** Anti-HB3var03 CIDRα1.4 IgG levels in serum. **b** EPCR-binding inhibition of pooled, purified IgG in  %. **c** Serum-IgG levels to 42 CIDRα1 protein variants not including the immunogen variant HB3var03 measured in 3–5 mice. **d** EPCR-binding inhibition of 33 EPCR-binding CIDRα1 protein variants (also excluding HB3var03). All for mice immunized with catCIDRα1.4 or CIDRα1.4cat on VLP or as soluble antigen in Freund’s incomplete adjuvant. Box plots showing median reactivity with 25th and 75th percentiles, upper and lower adjacent values and outliers. *Indicate statistically significant difference, P = 0.03 and P = 0.05 for catCIDRα1.4 and CIDRα1.4cat, respectively using Wilcoxon rank-sum test

Next, the reactivity of serum IgG to different CIDRα1 protein variants (N = 43), and the ability to inhibit binding between 34 of these variants and EPCR with total purified IgG, was tested using a Luminex based multiplex-system. Luminex microspheres coupled with individual CIDRα1 domains were mixed and used in assays measuring the amount of IgG binding each antigen (IgG level, Mean Fluorescence Intensity (MFI)) or the ability of EPCR to bind CIDRα1 on the microsphere in the presence of IgG (inhibition of EPCR-binding, Inhibition (%)). Figure 3 shows the antibody levels measured in serum from individual mice, and the inhibitory activity of purified total IgG pooled from animals receiving the same vaccine (both with serum collected 2 weeks after the third immunization). This analysis confirmed that the VLP vaccines induced higher levels of IgG to the cognate immunogen (HB3var03) than the vaccines with soluble antigen in Freund’s incomplete adjuvant (Fig. 3a, P = 0.03 and P = 0.05 for catCIDRα1.4 and CIDRα1.4cat, respectively; Wilcoxon rank-sum test). Furthermore, all vaccines induced IgG with high capacity to inhibit EPCR-binding of the cognate CIDRα1 immunogen (Fig. 3b).

**Fig. 4:**
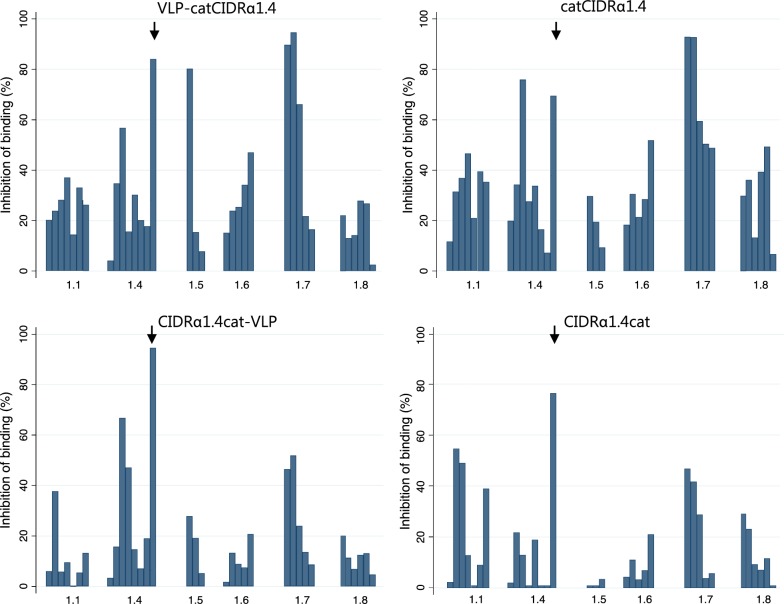
Inhibition of EPCR-binding of specific CIDRα1 domain variants. EPCR-binding inhibition (%) of 34 recombinant CIDRα1 domains by purified IgG pooled from mice immunized with VLP-catCIDRα1.4, catCIDRα1.4, CIDRα1.4cat-VLP or CIDRα1.4cat. Each bar represents one specific recombinant CIDRα1 domain variant annotated according to its domain subtype. Arrows indicate the HB3var03 variant used as immunogen. Order of CIDRα1 protein variants tested is shown as listed in Fig. 6a

When assessing the serum reactivity and EPCR-binding inhibitory potential of purified IgG to the panel of CIDRα1 domains not included in the vaccine (Fig. 3c, d), reactivity to and inhibition of specific variants varied considerably. However, there was no overall difference in these parameters between the VLP coupled and the soluble vaccines. When plotting the inhibition data for each specific CIDRα1 domain variant assayed (Fig. 4), inhibition was consistently observed for some domains. Overall, the CIDRα1 variants to which inhibitory antibodies were elicited were common to the four vaccines, albeit inhibition was somewhat more pronounced for IgG elicited by the catCIDRα1.4 antigens. If 40% inhibition was applied as a cutoff the VLP-catCIDRα1.4 vaccine induced IgG, inhibitingsix of 20 tested group A CIDRα1 variants (CIDRα1.4–7) and none of 13 CIDRα1.1/1.8 variants tested from the group B/A PfEMP1 (a.k.a. DC8 PfEMP1). IgG elicited by the soluble catCIDRα1.4 vaccine inhibited seven of 20 heterologous CIDRα1 variants from group A, and two of 13 CIDRα1.1/1.8 variants. Interestingly, in particular the binding of CIDRα1.7 domains, which are closest related to CIDRα1.4 used as immunogen, were inhibited. Raw data given in Additional File 1.

**Fig. 5:**
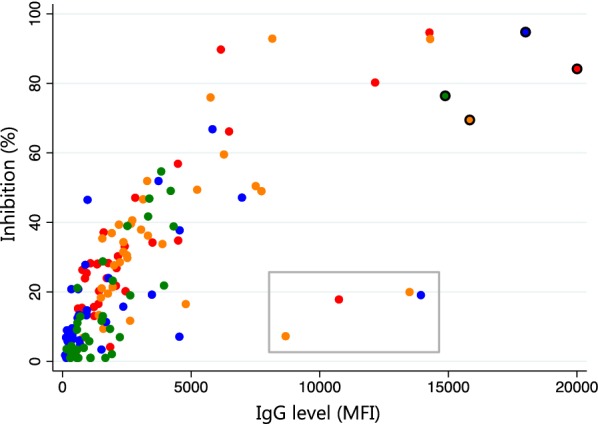
Correlation between anti-CIDRα1 IgG levels and EPCR-binding inhibitory capacity of CIDRα1 domains in pooled IgG purified from mice immunized with VLP-catCIDRα1.4. Each dot represents one EPCR-binding CIDRα1 domain and colors represent each of the four immunization schemes (34 domains × 4 immunization groups): VLP-catCIDRα1.4 (red), catCIDRα1.4 (orange), CIDRα1.4cat-VLP (blue), and CIDRα1.4cat (green). The immunogen CIDRα1.4 variant is outlined. The box includes indicates data from three CIDRα1.4_DD2var32 and one CIDRα1.4_1983-5 domain

There was a clear but not absolute correlation between IgG level to and the IgG binding inhibition of the single CIDRα1 domains (Ks = 0.67, P = 0.0001, Spearman’s rank-order correlation) (Fig. 5). For many domains, however, there was a considerable antibody recognition (MFI > 1000) without high inhibitory capacity. A particular high reactivity without strong inhibition was seen for two specific CIDRα1.4 variants (CIDRα1.4 1983–5 and CIDRα1.4_DD2var32). This may indicate that elicited antibodies targeted shared non-inhibitory epitopes of the domains or that the affinity of the elicited antibodies was insufficient to compete with EPCR.

**Fig. 6:**
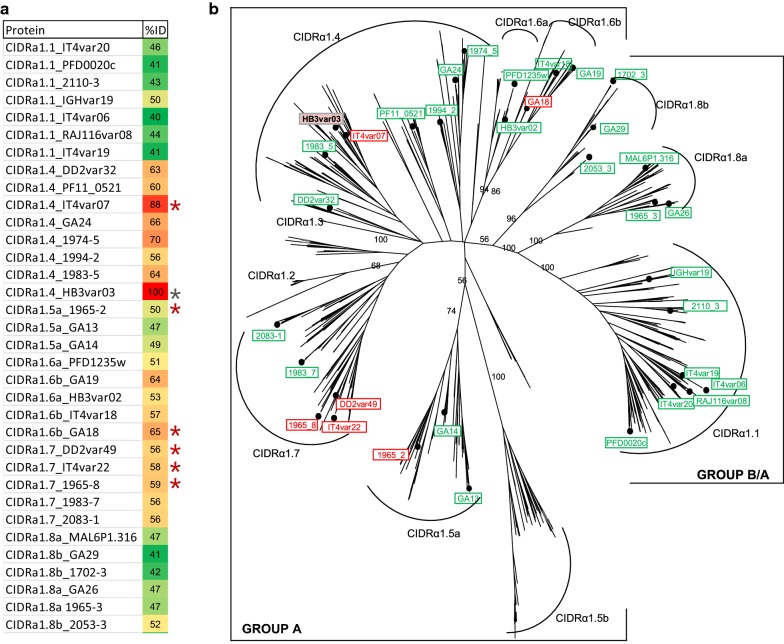
Sequence analysis of CIDRα1 domains. **a** Pairwise sequence similarity (19 kDa sequences) of the assayed CIDRα1 domains to HB3var03 CIDRα1.4. Blue (immunogen variant) and red asterisks (*) mark CIDRα1 domain variants inhibited > 40%. **b** Maximum likelihood tree (key bootstrap [N = 50] values are indicated on branches) of 885 CIDRα1 sequences (30 kDa) (generated in [26]). The 34 CIDRα1 variants tested for EPCR-binding inhibition (marked with boxes) are red if inhibited > 40% by total purified IgG from animals immunized with VLP-catCIDRα1.4 or for the cognate immunogen, blue. CIDRα1 variants not inhibited are green

Figure 6 shows the sequence relation between the studied CIDRα1 variants. Overall sequence similarity between immunogen and antigen was not clearly associated with antigen inhibition, albeit that the most similar domain, sharing 88% amino acids with the immunogen variant, was the strongest inhibited. No distinct amino acid sequence trait shared between inhibited CIDRα1 variants could be identified from sequence alignments (from visual inspection and with SigniSite, http://www.cbs.dtu.dk/services/SigniSite/). However, overall it was mainly CIDRα1 variants within group A, and in particular the CIDRα1.7 variants, that were inhibited.

## Discussion

Increasing evidence support that the interaction between the CIDRα1 domains of PfEMP1 and EPCR is key to the development of severe *P. falciparum* malaria [38]. Parasites causing severe malaria mainly express PfEMP1 binding EPCR and increasing proportions of EPCR-binding parasites appears to be associated with increase with severity of symptoms. No other PfEMP1 domain or trait shows this association [20, 21, 39–44], despite all PfEMP1 carrying additional domains capable of interacting with host receptors. This strong association may be tied to the observation that CIDRα1 binding to EPCR disrupts EPCR function, triggering endothelial inflammation and vascular leakage, characteristic for the pathology of severe malaria [45]. Children in malaria endemic areas develop immunity to severe malaria after relatively few infections coinciding with the acquisition of antibodies to CIDRα1 [9, 46]. For these reasons, CIDRα1 is considered an important target for vaccines protecting against clinical and particularly severe malaria [47].

However, the challenge to vaccine development is the antigenic diversity of the CIDRα1 domains. Based on amino acid sequence, the domains can be divided into six subclasses (1.1, and 1.4–1.8), each of which contain many different sequence variants. Despite the considerable amino acid variation, the protein structure and molecular basis of the interaction with EPCR is maintained across CIDRα1 domains [26]. Immunity to severe malaria is acquired relatively fast, which suggests that natural infection can induce IgG, targeting a relatively broad panel of CIDRα1 types. This notion is supported by the fact that IgG purified from malaria-exposed children and by affinity for a single CIDRα1.1 variant, can inhibit the EPCR-binding of distant CIDRα1.4 variants and vice versa [26]. The complexity of these antibody pools remains unknown. However, as short 63-mer peptides spanning the EPCR binding site was used for affinity purification in these studies, the data suggest that inhibitory antibodies interact with similar epitopes at or near the EPCR-binding site of different CIDRα1 variants [26].

In the present study, CIDRα1.4 immunization readily produced antibodies inhibiting the EPCR-binding of the CIDRα1.4 variant present in the vaccines as well as other CIDRα1 variants typically within the same subgroup as the immunogen. This was also seen in previous studies [27, 28], where animals were vaccinated with soluble recombinant CIDRα1 domains or live attenuated adenovirus inducing in vivo CIDRα1 protein secretion. In these studies, induction of cross-reactive antibodies was most clearly demonstrated by vaccination using two CIDRα1.1 domains resulting in antibodies reactive to 11 of 14 group B/A (CIDRα1.1 and CIDRα1.8) antigen variants [27]. The group B/A PfEMP1 are encoded by a set of chimeric *var* genes originating from group A and B *var* gene recombination events 5′ to the CIDRα1 sequence. Due to the opposite directions of transcription of group A vs. B and B/A *var* genes, these *var* gene subfamilies are likely to have evolved separately. Within group A PfEMP1, CIDRα1 domains have diverged into groups, with CIDRα1.4 and 1.7 variants being the closest related. Within subgroups CIDRα1 domains are typically 65–75% pairwise identical [16]. Between group A and B/A, CIDRα1 variants are on average 46% pairwise identical. The data presented here, indicate that immunization with a group A CIDRα1 mainly elicited cross-binding inhibitory antibodies against other group A CIDRα1 variants. This suggests that group B/A and group A CIDRα1 domains, to a certain extent comprise two separate serogroups. It was not possible from the present data to identify shared specific epitopes responsible for recognition and inhibition across variants.

Vaccines based on VLP-conjugated antigens are very effective in inducing long-lived functional antibodies. The best examples are the HPV vaccines, which after two immunizations elicit IgG maintained at stable levels for decades [29, 30]. Recently, methods that enable coupling of complex antigens to pre-formed VLP have been developed, employing the SpyTag/SpyCatcher conjugation system [31, 33, 34]. Employed in mice, these VLP vaccines have induced high and stable IgG levels against microbial antigens, including malaria antigens [48–51]. The VLP system secures a dense presentation of the vaccine antigen keeping an identical orientation on a rigid virus particle. The dense and repetitive presentation of antigens in their native conformation can override B cell tolerance and thus may alter not only the strength and longlivity of the response, but potentially also the epitopes targeted. In the case of CIDRα1 antigens, VLP presentain could therefore potentially alter the breadth of the immuneresponse to a broader reactivity across the protein family.

Here, the VLP-conjugated CIDRα1.4 vaccines induced an IgG response, which was higher and more rapid than the corresponding soluble vaccines formulated in Freund’s incomplete adjuvant. The EPCR-binding inhibitory effect across diverse CIDRα1 domains was similar for antibodies elicited by the VLP-based and the soluble CIDRα1.4 vaccines. This is important as Freund’s incomplete adjuvant cannot be used in humans, whereas the VLP platform is expected to be approved for human use and is likely be able to induce high tittered long-lasting immune responses in humans [52].

Immunizations with CIDRα1.4 fused to SpyCatcher N-terminally appeared to induce somewhat higher levels of inhibition than CIDRα1.4 with a SpyCatcher fused C-terminal. The C-terminal of CIDRα1 is positioned near the EPCR-binding site [26], and it is possible that a C-terminal fusion of a SpyCatcher adversely affected induction of antibodies to epitopes near or at the EPCR binding site. Overall, the VLP-presentation of CIDRα1 did not improve the reactivity across the CIDRα1 family, and it is likely that only a fraction of the elicited antibodies are functional. Additional studies testing inhibition of homologous native CIDRα1.4 PfEMP1 may further elucidate the relevance of the differences seen in EPCR binding inhibition by IgG elicited by the antigens presented on or off the VLP.

## Conclusions

This study showed that immunization with VLP-conjugated CIDRα1.4 antigens induced a rapid and strong IgG response capable of inhibiting EPCR-binding of CIDRα1 proteins belonging to different CIDRα1 subgroups. Data from this and previous studies suggest that vaccination with a limited combination of antigens can induce an antibody response covering most EPCR-binding CIDRα1 variants. Future studies including in vitro studies using natively expressed PfEMP1, are needed to clarify how many variants are required to obtain broad coverage across the CIDRα1 protein family and if such combinations can be achieved through display of multiple antigens on vaccine platforms, such as VLP vaccines, most likely to induce potent immune responses in humans.

## Supplementary information

**Additional file 1.** Raw data file. Data of IgG reactivity (MFI) and EPCAR binding (MFI) with and without presence of IgG given along with calculated level of inhition (%).

## Data Availability

All data generated or analysed during this study are included in this published article.

## Abbreviations

EPCR: Endothelial protein C receptor
PfEMP1: *P. falciparum* erythrocyte membrane protein 1
ICAM1: Intercellular adhesion molecule 1
CIDR: Cysteine-rich interdomain region
DBL: Duffy binding-like
VLP: Virus-like particle
